# Prediction of Obstetric Patient Flow and Horizontal Allocation of Medical Resources Based on Time Series Analysis

**DOI:** 10.3389/fpubh.2021.646157

**Published:** 2021-10-14

**Authors:** Hua Li, Dongmei Mu, Ping Wang, Yin Li, Dongxuan Wang

**Affiliations:** ^1^Department of Abdominal Ultrasound, First Affiliated Hospital of Jilin University, Changchun, China; ^2^School of Public Health, Jilin University, Changchun, China; ^3^Department of Clinical Laboratory, First Affiliated Hospital of Jilin University, Changchun, China

**Keywords:** forecast, patient follow, DRG, seasonally, trend, medical costs

## Abstract

**Objective:** Given the ever-changing flow of obstetric patients in the hospital, how the government and hospital management plan and allocate medical resources has become an important problem that needs to be urgently solved. In this study a prediction method for calculating the monthly and daily flow of patients based on time series is proposed to provide decision support for government and hospital management.

**Methods:** The historical patient flow data from the Department of Obstetrics and Gynecology of the First Hospital of Jilin University, China, from January 1, 2018, to February 29, 2020, were used as the training set. Seven models such as XGBoost, SVM, RF, and NNAR were used to predict the daily patient flow in the next 14 days. The HoltWinters model is then used to predict the monthly flow of patients over the next year.

**Results:** The results of this analysis and prediction model showed that the obstetric inpatient flow was not a purely random process, and that patient flow was not only accompanied by the random patient flow but also showed a trend change and seasonal change rule. ACF,PACF,Ljung_box, and residual histogram were then used to verify the accuracy of the prediction model, and the results show that the Holtwiners model was optimal. R2, MAPE, and other indicators were used to measure the accuracy of the 14 day prediction model, and the results showed that HoltWinters and STL prediction models achieved high accuracy.

**Conclusion:** In this paper, the time series model was used to analyze the trend and seasonal changes of obstetric patient flow and predict the patient flow in the next 14 days and 12 months. On this basis, combined with the trend and seasonal changes of obstetric patient flow, a more reasonable and fair horizontal allocation scheme of medical resources is proposed, combined with the prediction of patient flow.

## Introduction

In recent years, with the implementation of China's two child policy, the number of pregnant women is increasing rapidly. In addition, with the improvement of people's living standards, patients' pursuit of high-quality medical resources is also rising (1). For hospitals that can provide high-quality medical services and higher medical skills, the number of patients they receive each year is increasing year by year (2). In view of the ever-changing flow of patients received by hospitals, how the government and hospital management plan and allocate medical resources has become an important problem that requires urgent attention (3).

Hospital patient flow, congestion, and long waiting time are considered to be increasingly serious worldwide problems in the field of health care (4). Improper treatment of the above problems by hospital management can directly affect the patient's medical experience, and even hinder people from being admitted to hospital due to the lack of hospital reception capacity, and at the same time, result in an overload of work for medical staff (5). By accurately predicting the time and scale of patients' visits, managers can make better use of their limited resources (6). Thus, improving the utilization rate of medical resources and improving the medical experience of patients.

In recent years, the prediction of hospital patient flow has attracted more and more attention from business and academic circles (7, 8). Patient flow prediction is based on historical patient arrival and flow data, using a time series analysis prediction model to predict the arrival time and flow of patients in the future (9). When hospitals perform poorly in emergency patient flow handling, they seriously affect the medical experience of emergency patients (10). Patients sometimes have to bypass the nearest emergency hospital and take other emergency hospitals, which indirectly leads to an increase in the incidence rate and mortality rate (11). There is a commitment to the study of patient flow prediction in the literature on emergency room services (12). Time series techniques such as seasonal autoregressive integral moving average and generalized autoregressive conditional heteroscedasticity were used to predict patient flow (13, 14), providing decision support for management decision-making level, and providing more emergency patients with more urgent medical experience. Jones et al. (15) compared the performance of the regression index, smoothing, seasonal ARIMA, and the artificial neural network in emergency flow prediction. Wargon et al. (16) used the SPSS software package to determine a regression model and used three-year training data from four different hospitals to evaluate its one-year prediction performance. Reis and Mandl (17, 18) used the SAS software package to fit the ARIMA model into the historical data of the emergency department of the hospital in the past 10 years, and the model was verified accurately according to the last 2 years of the data set. A study by Liu and Ma (19) outlined that rural medical services play an important role in protecting and promoting the health of rural populations, and studied the degree of satisfaction with rural medical services. The most satisfied areas outlined by outpatients and inpatients related to the attitude of medical services and the explanation of disease conditions. Waiting time and medical expenses were the areas with which outpatients and inpatients were dissatisfied. To achieve forward-looking bed management, Jilani et al. (20) and Wimsatt (21) put forward the fuzzy time series to predict the number of visits of emergency patients, and used historical data to predict the patient flow of emergency departments in four hospitals in the United Kingdom. This method is more accurate and does not need any seasonal and periodic adjustment.

In terms of medical resource allocation, DRG has experienced more than 30 years of development. There is a growing interest in using DRG payment to repay hospitalization expenses worldwide. Many scholars have studied the DRG method. The purpose of this method is to change the previous retrospective payment method based on medical income to the settlement method based on medical output and make reasonable compensation for the medical resources consumed by medical institutions in the process of diagnosis and treatment through the packaging mode, so as to reduce the unreasonable growth of health expenses.

Tan et al. (22) put forward a medical and health care compensation method combined with activity weight, which allocates funds to hospitals according to the number and combination of clinical activities. This method is completely based on the compensation classification method of Australian refining DRG. Each refined DRG is assigned a weighted activity unit and converted to cost value to determine the annual ongoing funding allocation for each hospital. Australian public hospitals operate according to the fine diagnosis related group (AR DRG) (23) and separate them into specific DRG groups according to medical diagnosis or surgical procedures, patient age, separation method, clinical complexity, and complications, to effectively ensure the efficiency and fairness of hospital resource allocation. Chien et al. (24) then studied the impact of the DRG payment scheme on low value preoperative examination services. Through differential difference analysis, it was concluded that chest X-ray, echocardiography and blood pressure test, and other low-value examinations have decreased. The study concluded that financial incentives alone may not be enough to reduce the provision of low-value medical care.

According to the complexity of treatment and the impact of treatment time on the cost of treatment, Endrich et al. (25) have proposed the DRG scheme for newborns. Most of the mixed grouping schemes only classify newborns according to their birth weight. This method is used to determine the threshold definition based on the analysis of the variation point of birth weight and gestational age, and carry out detailed DRG classification. Bertoli and Grembi (26) studied the impact of political factors on DRG. After the implementation of budget restrictions, when there are doctors among politicians, the average level of DRG decreases. These analysis results show that DRG standardized price systems are not immune to political pressure. Vuagnat et al. (27) studied the impact of the implementation of a case-based payment system on postoperative readmission. The results showed that the rate of readmission in the private and public sectors increased relatively steadily and did not seem to be affected by the case-based payment system. Panagiotopoulos et al. (28) evaluated the Greek version of the DRG compensation system, and compared the DRG price with the average actual cost of each group of research cases, and evaluated the difference between the average length of DRG stay and the actual length of stay. Then a new DRG price calculation model is proposed, which is based on the actual cost calculation process of each year, which is composed of relative weight factors and benchmark price.

Based on the above research on patient flow prediction and DRG resource allocation, this paper proposes a method of patient flow prediction and the horizontal allocation of medical resources combined with time series analysis, to provide decision support for government and hospital management. The main contributions are as follows: using the time series analysis model to analyze and predict the obstetric patient flow, we analyze the trend and seasonal changes of obstetric patient flow, and predict the patient flow in each month in the next year. The trend and seasonal changes of obstetric patient flow were then studied, and a more reasonable and fair horizontal allocation scheme of medical resources was proposed combined with the prediction of patient flow.

## Data and Methods

### Data

The historical patient flow data from January 1, 2018, to February 29, 2020, in the Department of Gynecology and Obstetrics at the First Hospital of Jilin University, China, were used as the training set. We did not use data from before 2018 due to the lack of data. However, due to the outbreak of the new crown epidemic, the hospital has imposed a patient flow restriction on the obstetrics department, that is, the data after March 2020 are not of trend and seasonal variation, and the data in this period were not used. In order to realize the analysis and prediction of monthly patient flow, the data were classified and summarized to achieve the monthly patient flow, and then 26 data points were obtained.

### Smoothing of Time Series

**Figure 1:**
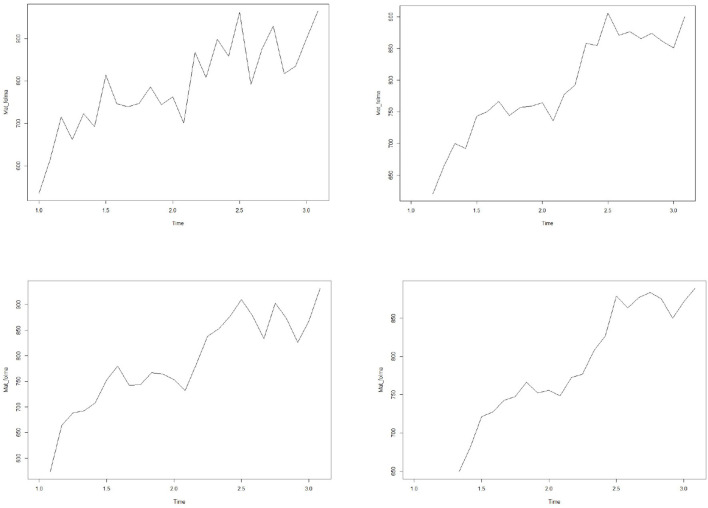
Smoothed time series of patient flow.

Time series data sets usually contain significant random or error data. In order to analyze the trend rule in the data, we hope to delete these random fluctuations by drawing a smooth curve. The simplest method is to draw smooth curves by SMA function. The four graphs in Figure 1 represent the corresponding smooth curves of orders 1, 2, 3, and 5 respectively. It can be seen from the figure that these data have obvious seasonal and trend change rules.

### Data Decomposition

In the time series data with seasonal factors, the data can be decomposed into trend factors, seasonal factors, and random factors. The trend component can capture the change of future cycles, the seasonal component can capture the seasonal change of a cycle, and the random / error component can capture the random change that cannot be explained by the trend or seasonal change.

**Figure 2:**
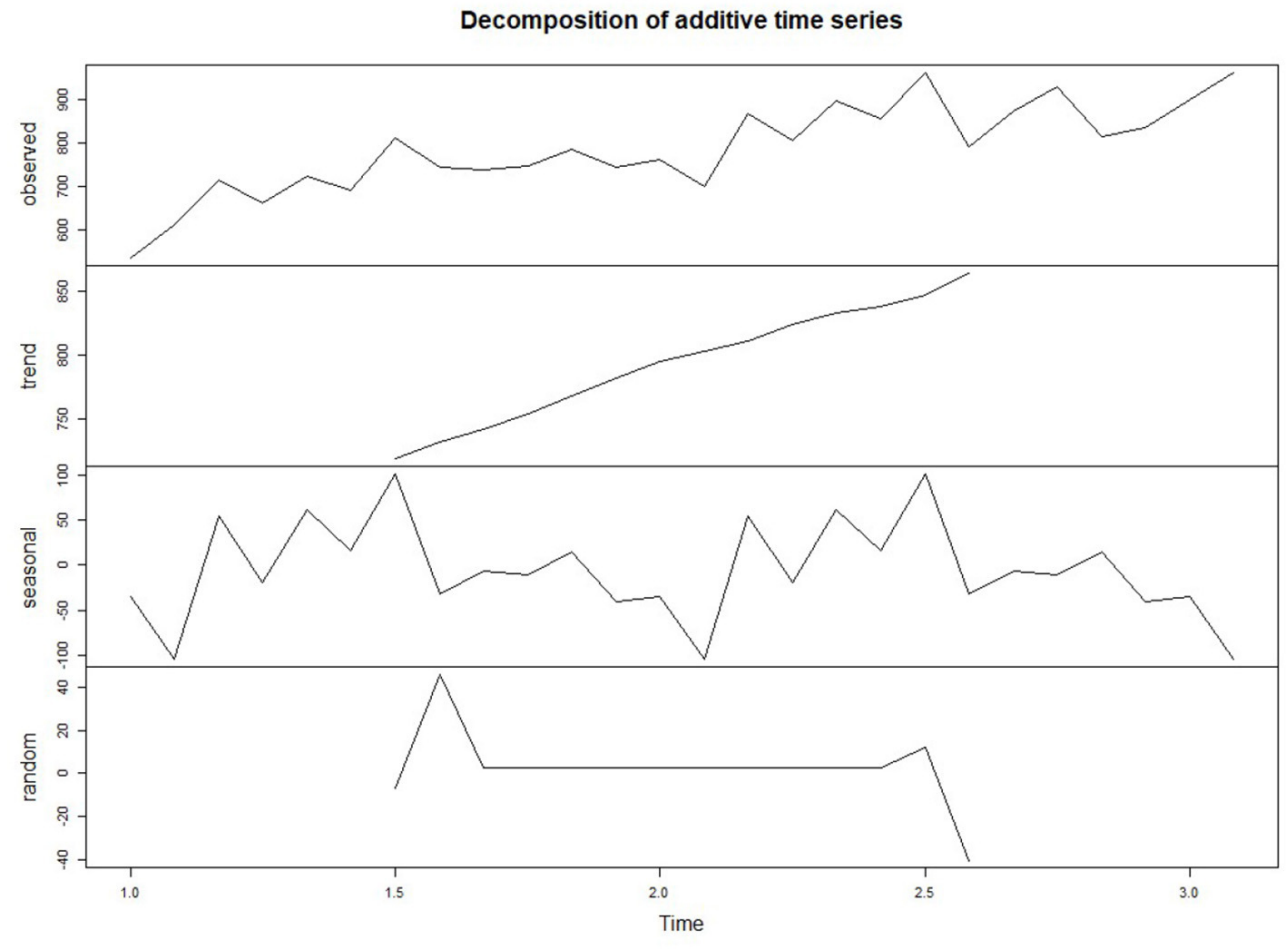
Decomposed patient flow time series diagram.

The decomposition function is used to decompose the time series data, and the decomposed data graph is shown in Figure 2. It can be seen from the seasonal part of the figure that the flow of pregnant women patients has a peak in autumn and a trough in winter. In the trend part, the number of pregnant women outpatient services increased periodically with time. With time, the random fluctuations in the time series seem to be roughly unchanged, showing a normal distribution.

## Results

### Monthly Flow Forecast

#### Prediction Model

**Figure 3:**
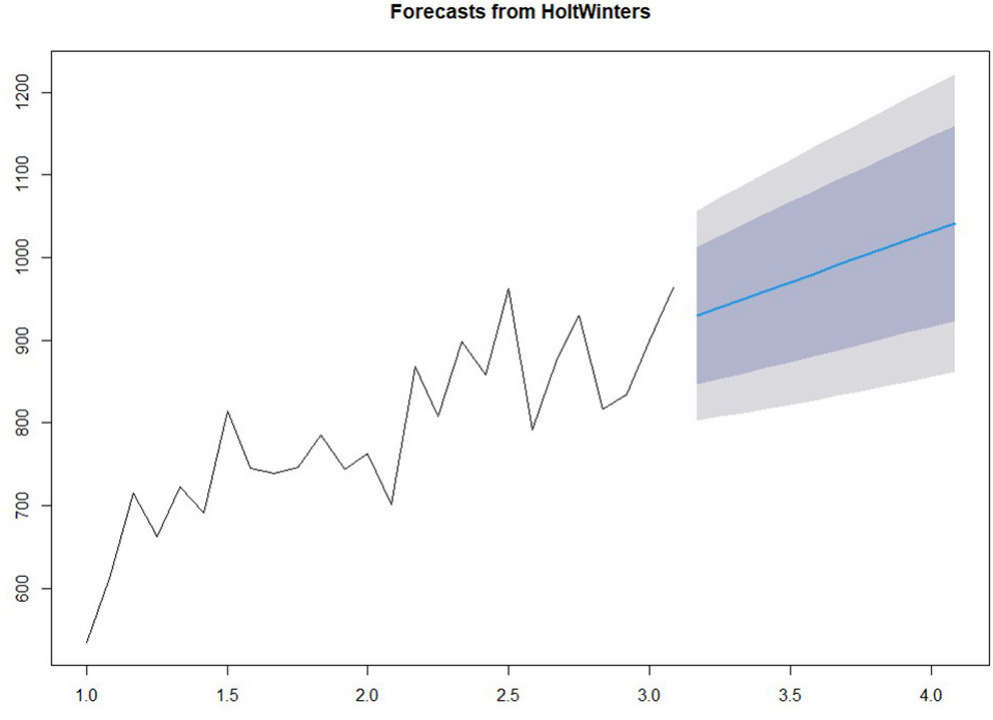
Prediction of patient flow.

We use the HoltWinter model in the forecast package to fit a simple exponential smoothing prediction model to predict the number of pregnant women outpatient services in the next year. The prediction results are shown in Figure 3. Among them, light gray and gray are the predicted values corresponding to 80 and 95% confidence intervals respectively.

#### Accuracy

**Figure 4:**
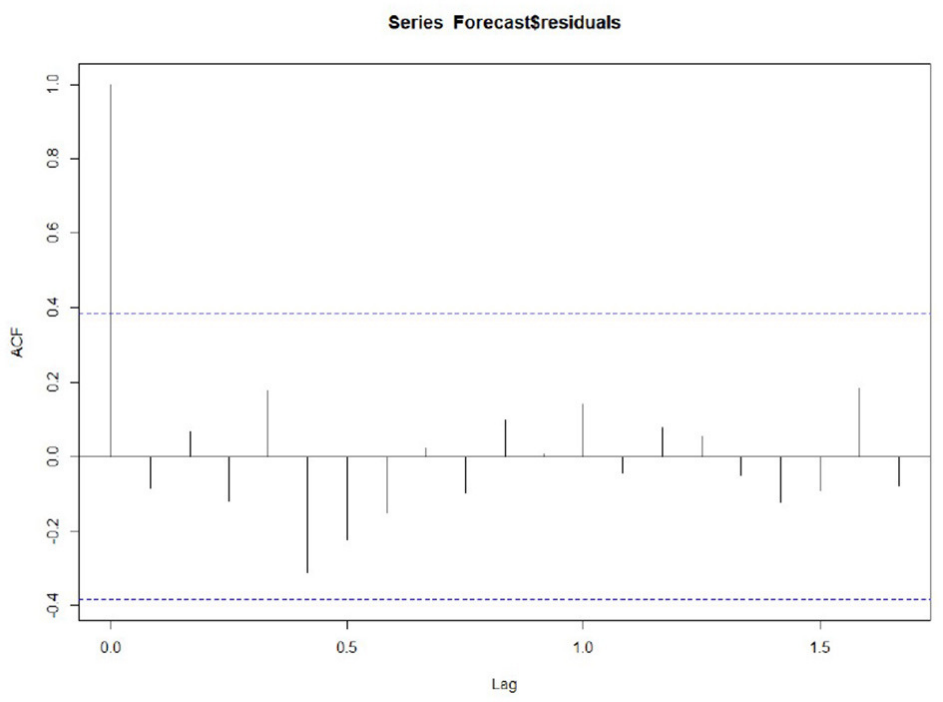
Autocorrelation.

**Figure 5:**
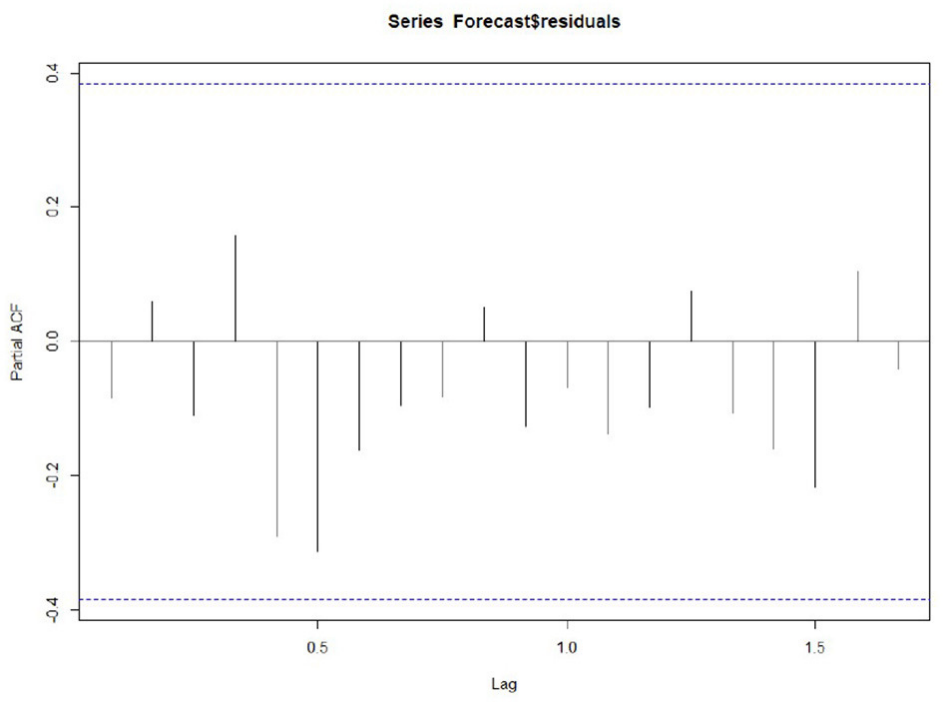
Partial correlation graph.

**Figure 6:**
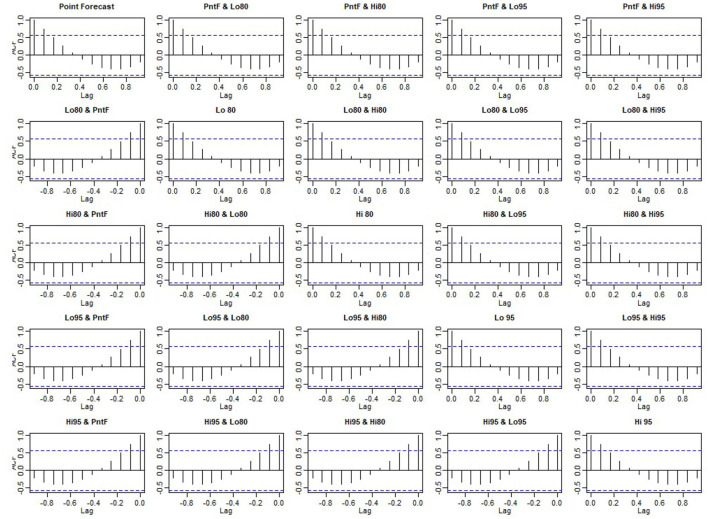
Detailed autocorrelation diagram.

Whether the prediction model can continue to be optimized is determined by whether there is a correlation between the prediction errors of continuous prediction, that is, if there is no correlation between the prediction errors, the prediction model cannot be further optimized. ACF,PACF,Ljung box were used most to analyze the correlation. Among these functions, the ACF in R is used to calculate the error of the correlation graph. To specify the maximum order you want to see, you can use the Lag.max Parameter. In this case, the correlation diagrams of the 1–20 order delay of the flow prediction error of the maternal patients are calculated, as shown in Figures 4, **6** below. Figure 4 indicates that the autocorrelation system does not touch the confidence limit in order 1–20. It can be seen from Figure 5 that the partial correlation coefficients do not touch the confidence limit. Detailed ACF cases for 80 and 95% are given in Figure 6.

To test whether the non-zero correlation is significant when the lag is 1–20 in order, we used the Ljung box test and passed the test in the R software Box.tex Function implementation. The maximum order can be achieved by setting the Box.tex lag parameter. The Ljung box test statistic is 17.4, and the p-value is 0.6. This is not enough to prove (almost no evidence) that the prediction error in the sample is non-zero autocorrelation in the lag order of 1–20.

**Figure 7:**
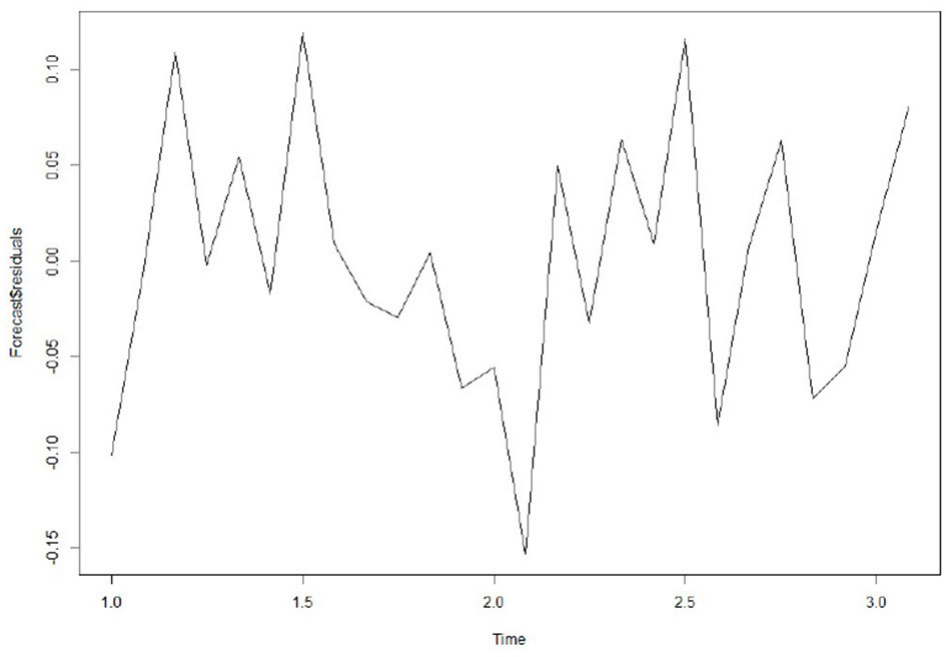
Prediction residual diagram.

**Figure 8:**
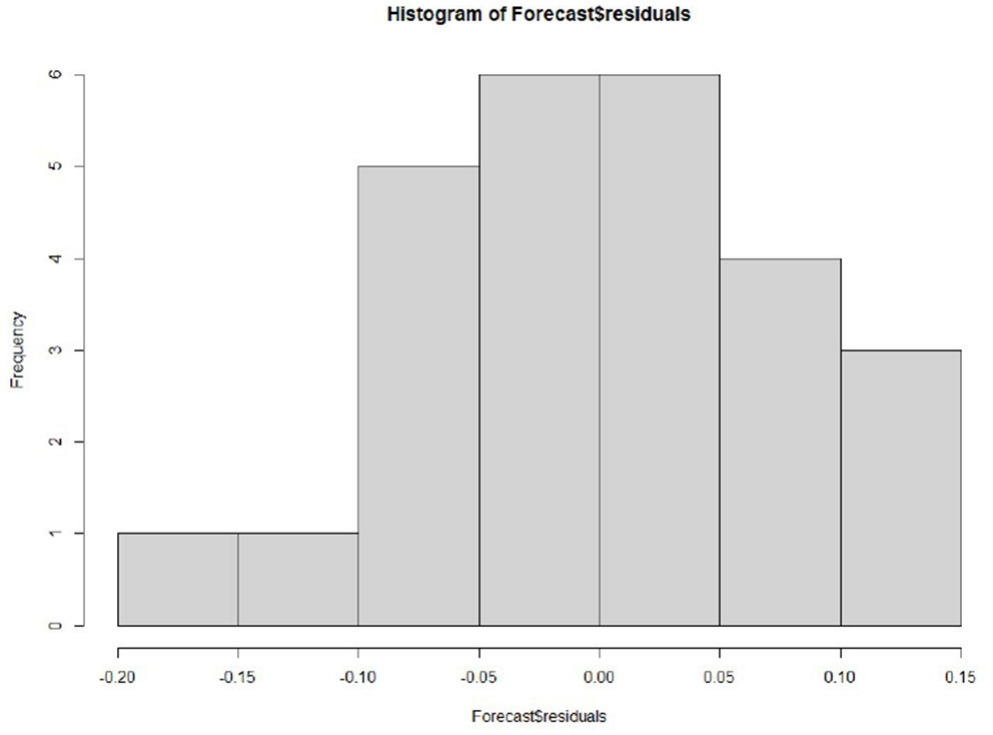
Histogram of prediction residuals.

In addition, to ensure that the prediction model is the best, it is also a good way to check whether the prediction error has a normal mean of 0 and constant variance. For each test point, the prediction error can only be calculated by subtracting the predicted value from the observed value. Therefore, the prediction errors of the existing time series can only be calculated. To test whether the variance of the prediction error in the whole sequence remains unchanged, that is, the normal distribution of service value-added, we draw the prediction error graph and the prediction error distribution histogram of the normal curve in a period of time. Figure 7 shows that the random fluctuation above the time series tends to be a constant with the increase of time; that is, it is stable in the mean value and variance. As can be seen from Figure 8, the histogram shows that the distribution of prediction error is ~0-centered and more or less normal distribution. Although it seems to be slightly to the right, the tilt is relatively small, so the prediction error is approximately distributed with a mean of 0.

Because the continuous prediction error seems to have no autocorrelation and partial correlation, and the prediction error seems to be a normal distribution, the mean value is 0 and the variance is constant, meaning the prediction model can accurately predict the patient flow.

### Daily Flow Forecast

#### Prediction Model

We used seven prediction models, such as XGBoost, SVM, RF, and NNAR, to predict the daily patient flow in the next 14 days. The basic principles of the seven models are introduced below.

The XGBoost (Extreme Gradient Boost) model is a special Gradient Boost decision tree that maximizes speed and efficiency (29, 30). XGBoost is essentially a tree-based approach combined with ensemble learning. The base tree is a classification regression tree. Similar to the local weighted linear regression algorithm, the tree-based regression algorithm is also a kind of local regression algorithm. By cutting the data set into pieces, each piece of data is modeled separately. But the difference is that a tree-based regression algorithm is a parameter-based learning algorithm. After training the model with the training data, once the parameters are determined, there is no need to change. A classification regression tree is a structure based on a decision tree, which can be used to solve both classification and regression problems. It is one of the first ten classical algorithms in the field of data mining.

XGBoost exports the enhancement tree by optimizing the objective function, which can be used to solve almost any objective function that can be written as a gradient. This includes things like ranking and Poisson regression, and random forests are difficult to implement. The XGBoost model is more sensitive to over-fitting if the data is noisy. Because trees are built in sequence, training often takes longer.

The SVM model (31) transforms the source data into a higher dimensional space, where each input vector forms a sequence of seed points with a time delay. These vectors are then combined as key samples in a special way that allows the regression hyperplane to define the data distribution, and calculate with specified precision. These calculations represent the sum of all the samples called the “kernel,” the integration function of the inputs. These functions can be linear or non-linear, and the guiding parameters affect the regression accuracy.

The advantages of SVM are as follows: it can deal with machine learning in the case of small samples, solve the problem of high-dimensional data, and avoid the problem of neural network structure selection and local minimum. This model has the following shortcomings: there is no universal solution for non-linear problems, so the Kernel function must be carefully selected. This model is also sensitive to missing data: when processing large-scale data, the memory resources of the computer are required to be high.

Random forest (32) is essentially a branch of machine learning called Ensemble Learning, which is a method of integrating many decision trees into a forest and using it to predict the final outcome. A Random Forest Model builds a forest in a random way. There are many decision trees in the forest, and there is no correlation between each decision tree in the random forest. Owning the forest, when a new input sample comes in, the decision tree in the forest makes its own judgment and determines to which category the sample should belong (for the classification algorithm). It then identifies which category is selected the most, and predicts which category the sample belongs to. A random forest can handle both quantities with attributes of discrete values and quantities with attributes of continuous values. In addition, the random forest can also be used for unsupervised learning clustering and outlier detection.

Model tuning is easier in a random forest than in XGBoost. In a random forest, we have two main parameters: the number of features to be selected for each node and the number of decision trees. Random forests are harder to configure than XGBoost. The main limitation of the random forest algorithm is that a large number of trees can slow down the algorithm for real-time prediction. For data containing categorical variables with different levels, the random forest favors those attributes with more levels.

NNAR is a model based on an artificial neural network and a prediction method based on a simple brain mathematical model (33). They allow complex non-linear relationships between response variables and their predictors. Neural networks can be considered as “neuron” networks organized by layers. The prediction variable (or input) constitutes the bottom layer, and the prediction (or output) constitutes the top layer. There may also be an intermediate layer containing “hidden neurons.” The simplest network does not contain a hidden layer, which is equivalent to linear regression. Once the middle layer with hidden neurons is added, the neural network will become non-linear.

NNAR can process temporal projections of large data sets. In addition, the prediction results are better for raw data with higher volatility. This model has many parameters, which start from random values during training, resulting in random factors in prediction. On the other hand, the network model needs to be trained many times with different random starting points, and the results are then averaged.

STL uses the Loess regression function to decompose the time series data into seasons, trends, and irregular components (34). What should be noted is that the prediction intervals ignore the uncertainty associated with the seasonal component. They are computed using the prediction intervals from the seasonally adjusted series, which are then reseasonalized using the last year of the seasonal component. The uncertainty in the seasonal component is ignored.

STL can handle any type of seasonal data, not just monthly and quarterly data. The smoothness of the trend cycle can also be controlled by the user. It is robust to outliers, that occasional outlier observations do not affect the estimation of trend periods and seasonal components. On the other hand, STL also has some disadvantages. In particular, it does not automatically handle changes in the trading day or calendar, it just provides additional decomposition.

If we combine autoregressive and moving average models, we will get a non-seasonal ARIMA model (35). In the autoregressive model, the linear combination of past values of variables is used to predict interesting variables in the future. The moving average model does not use the past value of the prediction variable in the regression but uses a past prediction error in the model similar to the regression.

The ARIMA model is very simple and requires only endogenous variables without recourse to other exogenous variables. The ARIMA model is recommended when the raw data does not fluctuate much. This model has the following disadvantages: it can only capture linear relations, but not non-linear relations. Temporal data is required to be stable, or stable through differential differentiation.

The Holt-winters method (36, 37) is a time series analysis and prediction method that is suitable for non-stationary series containing linear trends and periodic fluctuations. The exponential smoothing method is used to make the model parameters adapt to the changes of non-stationary series, and make a short-term prediction of the future trend. Holt winters method introduces winters cycle term based on the Holt model, which can be used to deal with the fluctuation behavior of a fixed cycle in time series such as monthly data, quarterly data, and weekly data.

Holt-winters is a method to predict time series by using cubic exponential smoothing. The cubic exponential smoothing algorithm can save the trend and seasonal information of time series data well. Through quadratic exponential smoothing, the overall trend information can be retained. The seasonal characteristics of time series are processed by cubic exponential smoothing. But this model is not suitable for long period series prediction.

**Figure 9:**
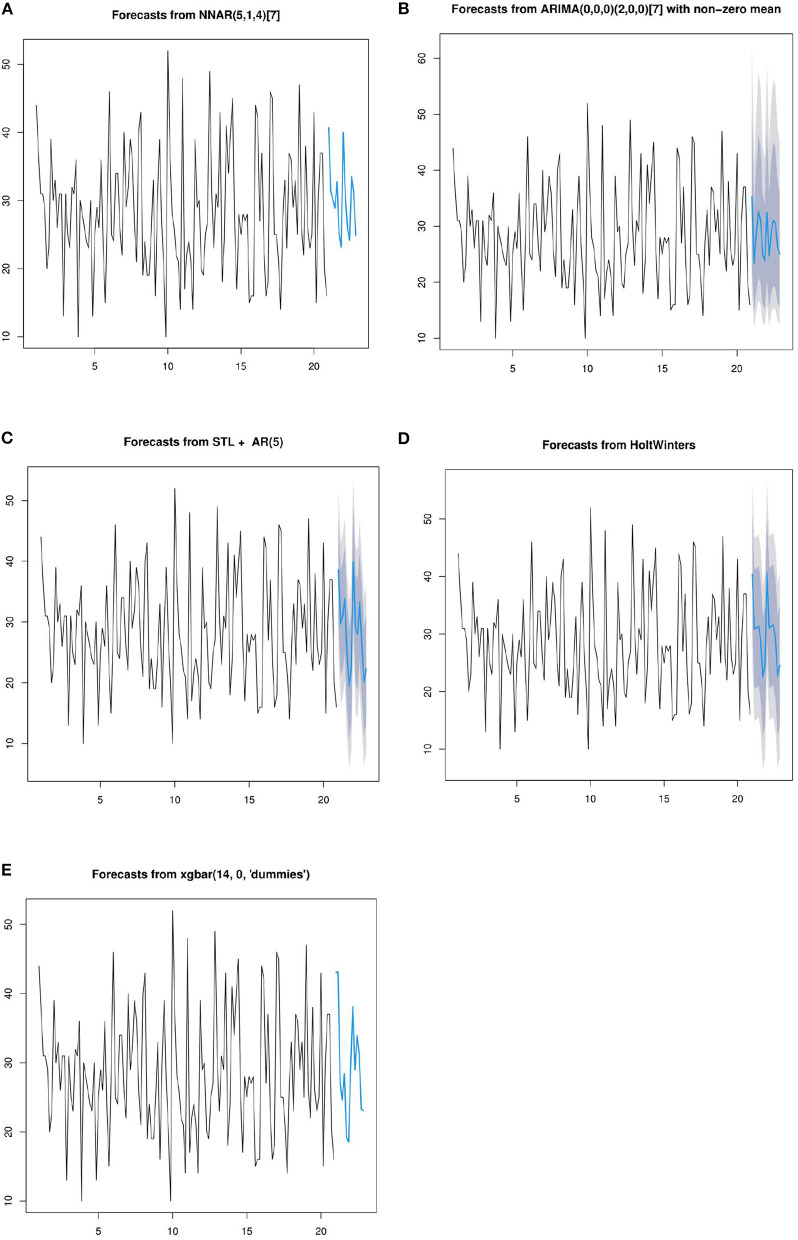
Forecasts from NNAR**(A)**, ARIMA **(B)**, STL **(C)**, HoltWinters **(D)**, and XGBoost **(E)**.

In the above section of this paper, the basic principles of seven prediction models have been introduced. The following seven models are used to predict the daily patient flow over the next 14 days. In order to facilitate the display of predicted data, the training data was selected from 20 cycles of data. First, periodize the time series data, and the number of days selected for the period is 7. The prediction results of the five methods are shown in the Figure 9.

**Figure 10:**
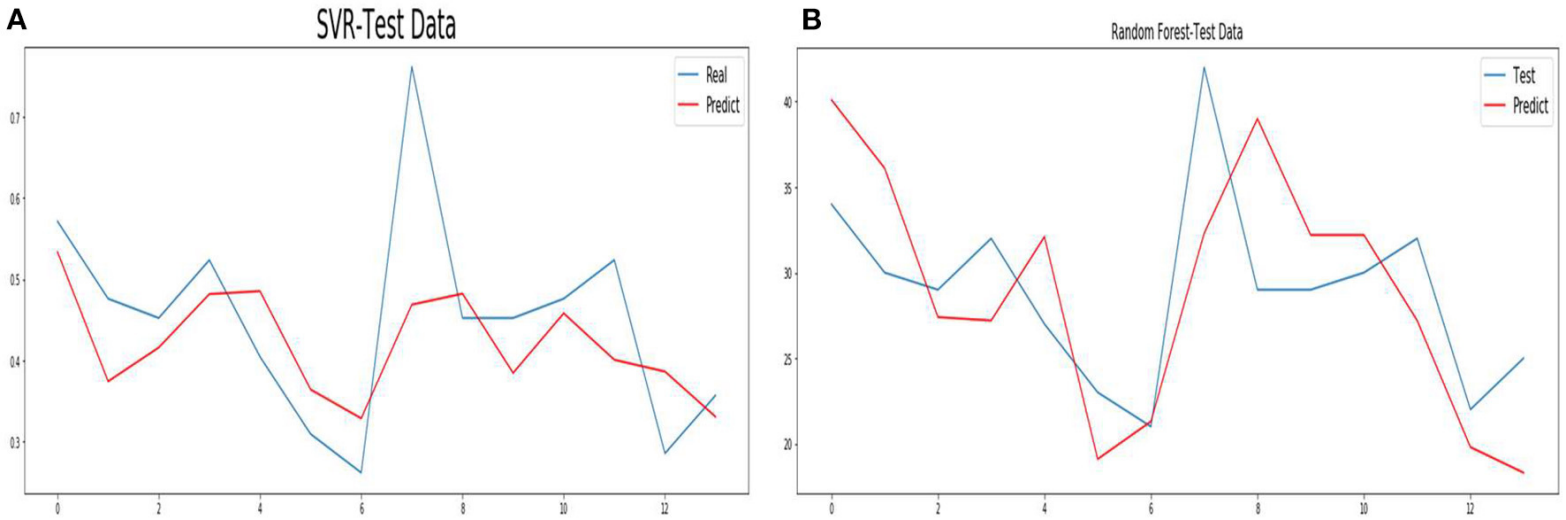
Forecasts from SVR **(A)** and Random Forest **(B)**.

To accelerate the speed of gradient descent in machine learning and improve the prediction accuracy, the time series data are normalized. SVM and RF methods conduct training and prediction on the normalized data, and the results are shown in Figure 10. For details of the hyperparameter values of the above seven models, see Appendix 2.

#### Accuracy

In this section, ME, RMSE, MAE, and other indicators are used to describe the accuracy of the seven methods. The meanings of ME, RMSE, MAE, and other parameters are shown in Appendix 1. The values of each parameter in the training set are shown in Table 1. The XGBoost method has the least value on each index, that is, the model fits the training data best. Second, the RF method has a MAPE value of 11.79, which has a better performance than the other methods. The value of the ARIMA and SVR methods in each parameter are large, indicating that these methods have the worst fitting on the training data. The maximum value of MAPE of the SVR method is 38.40; that is, the absolute difference of predicted data reaches 38.4% on average compared with all training data.

**Table 1:** Accuracy index of training data.

	**ME**	**RMSE**	**MAE**	**MPE**	**MAPE**	**ACF1**	**R2**
NNAR	0	4.87	3.81	−5.57	15.53	0.02	0.54
STL	−0.13	6.68	5.35	−7.39	21.64	−0.01	0.44
ARIMA	−0.14	8.38	6.91	−10.58	27.63	0.07	0.14
HoltWinters	0.65	8.09	6.50	−5.76	25.24	0.06	0.36
XGBoost	0.02	0.26	0.18	−0.11	0.65	−0.10	0.99
SVR	0.58	11.6	9.28	1.67	38.40	–	0.30
RF	0.21	4.02	3.24	−0.60	11.79	–	0.75

Table 2 shows the values of each parameter of the seven prediction models on the test set. From R2 and MAPE parameters, HoltWinters, and STL methods have higher prediction accuracy. The HoltWinters method has the best predictive performance with the highest R2 value of 0.833 of all the methods. The MPE value STL method is 1.95, while the MAPE value is 8.3, respectively. This indicates that most of the data predicted by this method are greater than the actual value. The MPE value of the HoltWinters method is −4.16, while the MAPE value is 6.68, respectively. This indicates that most of the data predicted by this method are smaller than the actual value. The MPE value of the ARIMA method is −0.02, while the MAPE value is 9.53, respectively. This indicates that half of the predicted data of the ARIMA method are greater than the actual value and half are less than the actual value.

**Table 2:** Accuracy index of testing data.

	**ME**	**RMSE**	**MAE**	**MPE**	**MAPE**	**R2**
NNAR	−1.2	4.11	3.28	−5.56	11.92	0.63
STL	0.47	2.8	2.36	1.95	8.30	0.83
ARIMA	0.44	3.8	2.77	−0.02	9.53	0.48
HoltWintes	−1.12	2.4	1.93	−4.16	6.68	0.83
XGBoost	−0.52	6.52	4.90	−2.02	15.98	0.57
SVR	1.74	5.8	4.64	6.42	18.47	0.43
RF	0.40	4.54	3.04	2.64	10.09	0.35

### Analysis and Discussion

The experimental results predicted by seven models are given above. In this section, combined with the advantages and disadvantages of the model and experimental data, the experimental results and the performance of the prediction model are further discussed. The HoltWinters and STL models achieve the best predictive performance. Their MAPE values are 6.68 and 8.3, and their R2 values are 0.833 and 0.832, respectively. The HoltWinters model has the best prediction performance, mainly because of its good processing of trends and non-stationary series of periodic fluctuations. This model is a method to predict the time series by using the cubic exponential smoothing method. Through quadratic exponential smoothing, the overall trend information can be retained. The seasonal characteristics of the time series are processed by the cubic exponential smoothing method. The prediction performance of the STL model is high mainly for the following reasons. This model can deal with any type of seasonal data, not just monthly and quarterly data, and has good performance for daily data. In addition, the model is very robust to outliers, and occasional outliers do not affect the prediction of trend cycles and seasonal components.

On the training set, XGboost and RF have the best fitting performance, with mAPE of 0.65 and 11.79 respectively. However, on the test set, the prediction performance is poor, and their R2 values are 0.57 and 0.35, respectively. The actual data of the hospital are used in this paper, and there is much noise in the data. XGBoost and RF models are sensitive to noise data during training, which leads to over-fitting of the model. SVR model has poor fitting performance in training data, and its MAPE value is 38.4. Its mAPE and R2 values are 18.47 and 0.43, respectively. This is mainly because SVR does not use the appropriate kernel function. Time series is a non-linear problem, so the method of choosing the kernel function has a great influence on model prediction performance. In addition, the model is sensitive to noise data. The MAPE and R2 values of the ARIMA model are 9.53 and 0.48, respectively. The actual data used in this paper fluctuated. When using the ARIMA model for prediction, the model cannot predict time series fluctuation data well, which leads to the model not achieving good performance.

## Discussion

This section of the paper first discusses the research status and shortcomings of DRG resource allocation methods at home and abroad, and then explores the progress and problems of medical insurance compensation in China. Then, according to the analysis and prediction method of patient flow, decision support for the government and hospital management is discussed. Finally, the limitations of the proposed method are analyzed.

### DRG Status and Deficiencies

The DRG payment method shortens the length of hospital stay but brings increasing medical expenses and medical costs. Its impact on health care and health outcomes is controversial, especially in low- and middle-income countries. Policymakers should carefully consider each component of the DRG payment design according to policy objectives (38) so as to allocate medical resources efficiently and reasonably (39).

Clinical records ultimately determine the future funding of our healthcare system. Proper communication and education between medical staff and hospital coding staff are essential to ensure accurate documentation and accurate AR-DRG coding and to achieve the best and the most reasonable compensation in DRG mode. Lisbeth et al. (40) have studied whether the introduction of the DRG system affects the number of secondary diagnoses. During the study period, hospitals were divided into two groups: those with DRG payment systems and those without DRG systems. In all regional hospitals, the number of coded secondary diagnoses has increased, but the number of secondary diagnoses per case has also increased. After the implementation of the expected payment system, the secondary diagnoses of the hospitals have increased greatly. Aiming at the problem of misclassification frequently occurring in DRG, Suleiman et al. (41) have proposed the introduction of expert conjecture combined with the Bayesian model to improve the occurrence of DRG errors and significantly improve the efficiency of clinical coding review.

DRG payments may be slightly more efficient, but they face upgrades in medical record codes and damage the quality and fairness of medical care (42). Damage to health care rights and interests has been reported, especially for patients who are not supported by this payment plan (43). Zou et al. (44) evaluated the impact of DRG on health care and health outcomes in China and comprehensively described the expenditure, efficiency, quality, and fairness of health care. Montefiori et al. (45) studied the DRG program in relation to newborns. The results show that the efficiency of the DRG system is low. The cost of the same DRG patient varies greatly, and even though the cost of the patients with very low birth weight is about twice the reimbursement amount stipulated by the policy; when the newborn is full-term, the cost is 20,000 euros less than that reimbursed by DRG.

Kim et al. (46) have studied the performance of DRG based compensation for medical and health care expenditure. Payment based on DRG shortens the hospitalization time, and also changes the behavior of doctors collecting DRG code from outpatient, and increases medical expenses and medical costs continuously. To some extent, there is no overall budget for national medical insurance. How to plan the DRG global budget is an important problem that needs to be solved in further research.

### Current Situation of China's Medical Insurance

Since the “new medical reform policy” was introduced in 2009, with the progress of medical reform year by year, the reform of medical security has achieved great results in many aspects, especially in terms of reimbursement content, reimbursement proportion, payment mode transformation, serious illness reimbursement systems, remote medical treatment systems, and the participation of commercial insurance. The new medical reform puts forward the short-term goal of effectively alleviating the difficulty and high cost of medical treatment, and the long-term goal of establishing and improving the basic medical and health system covering urban and rural residents, and providing safe, convenient, effective, and inexpensive medical and health services for the masses.

With the gradual development and improvement of medical security systems, the goal of universal medical insurance is possible, and the number of beneficiaries has been increasing in recent years. Because medical security is closely related to people's daily life, it occupies an extremely important part of the social security system. As the core resource of sustainable development of medical security, strengthening the supervision of basic medical insurance funds is of great significance to ensure the safe and sustainable operation of medical insurance funds.

To effectively curb the unreasonable growth of medical expenses and change the situation of “relying on drugs to support doctors,” China has accelerated the pace of medical and health system reform. In January 2017, the National Development and Reform Commission, the Health and Family Planning Commission, and the Ministry of Human Resources and Social Security jointly issued a notice on promoting DRG payment, requiring all localities to further promote DRG payment reform, mainly to include gradually expanding the scope of DRG payment, reasonably determining specific diseases and charging standards, solidly completing the connection of DRG payment, and earnestly implementing these various reform policies.

The direction of China's medical reform is to implement DRG payment in a well-rounded way. From the hospital level, it is imperative to carry out DRG cost accounting. DRG cost accounting calculates the cost of diagnosis and treatment by superposition of medical project costs, drug costs, and separate charging material costs for the treatment of a single disease. Practice shows that DRG cost accounting can not only reduce the cost and optimize the diagnosis and treatment process, but also provide data support for government departments to formulate reasonable disease costs and promote the formation of cost-oriented operation mechanisms. Therefore, the introduction and strengthening of the DRG accounting method is key to furthering cost accounting work in hospitals under the situation of new medical reform, especially under the reform of the DRG payment mode.

### Horizontal Allocation of Resources

As the medical center of Jilin Province in Northeast China, the first hospital of Jilin University ranks first in Jilin Province in terms of medical technology and abundant medical resources. In Jilin Province, medical institutions are divided into three categories: primary medical institutions, regional medical institutions, and tertiary medical institutions. Most medical institutions, especially regional and tertiary medical institutions, are public. The government has strict restrictions on the pricing of medical services. The loss of public hospitals is borne by the government financial department. All public medical institutions can enjoy the medical insurance policy.

At present, one of the main problems in the medical service system of Jilin province is that the total amount of medical resources is relatively insufficient and the utilization structure of medical resources is unreasonable. In this paper, we use the time series analysis model to analyze and predict the obstetric patient flow, analyze the trend and seasonal variation of obstetric patient flow, and predict the patient flow of each month in the next year. Based on the trend change, seasonal change, and patient flow of obstetric patients, a more reasonable and fair horizontal allocation plan of medical resources is given.

In this paper, the variation of patient flow trends was analyzed to provide decision support for government and hospital management. For diseases with an increasing tendency, the total amount of annual compensation can be increased according to the increase proportion. For diseases with a downward tendency, the total annual compensation will be reduced by floating. Then the total amount of compensation between different diseases can be adjusted horizontally. For hospital management, we can make a reasonable and efficient medical resource allocation plan according to the floating characteristics of patients every month.

The seasonal variation rule of patient flow obtained in this paper is helpful for hospital managers in providing more reasonable medical resource allocation plans for medical staff and patients. For patients, it provides patients with peak and low peak patient flow, thus providing a reference for patients in future hospitalization. For managers, it is conducive to resource allocation from the perspective of resource scheduling, to provide a better medical treatment experience for patients and guide medical staff to rest, study and work under the condition of limited resources. In the low peak period, some medical staff can take turns to take off work and study, thus providing medical staff with opportunities for rest and study, thus improving their life quality and medical quality. In the peak period, targeted medical staff should go to work in combination with the actual situation, which will improve the medical experiences of patients.

With the year-by-year increase of patient flow, the annual amount of medical insurance is less than the reimbursement amount of all patients in most cases. The existing medical insurance reimbursement system is based on the principle of first use first reimbursement, which often occurs at the end of each year that patients cannot reimburse or reimburse part of medical expenses. According to the monthly patient flow obtained by the prediction model, medical insurance compensation can be evenly covered for each patient in each month according to weight. Assuming that the predicted monthly patient flow is *M*_*i*_ (*i* = 1,2,…,12), the average hospitalization cost of each patient is *E*, and the annual reimbursement amount of medical insurance is *I*. then the total patient flow in the next year is *M*_1_ + *M*_2_ +… + *M*_12_, and the medical insurance cost to be paid is (*M*_1_ + *M*_2_+… + *M*_12_) ^*^ *E*. Based on the idea of horizontal resource allocation, according to the predicted patient flow, the amount of reimbursement for each person is *E* ^*^I / ((*M*_1_ + *M*_2_ +… + *M*_12_) ^*^E), that is, *I* / (*M*_1_ + *M*_2_ +… + *M*_12_). The above horizontal resource allocation scheme based on flow prediction enables every patient to use medical insurance fairly, avoiding the problem that patients cannot use medical insurance fairly at the end of each year.

### Limitations

The data used in this paper are only from a tertiary hospital in Jilin province, so the trend and seasonal variation of the analysis may not be suitable for the prediction of patient flow in all hospitals. In addition, in terms of patient flow prediction, the data used in this paper are <3 cycles, which also affects the accuracy of the prediction model in predicting future data. Finally, this paper only analyzes and predicts the flow model of patients in obstetrics, but it is also meaningful for other kinds of diseases. In future studies, it can be expanded to more hospitals and patients with more diseases. The analysis and prediction of patient flow in multiple hospitals and diseases will be more meaningful for the government and hospital management to provide decision support.

## Conclusion

At present, patients' demand for medical treatment is not only expected to obtain timely and effective medical services at a reasonable cost but also expected to enjoy a higher level of medical services at a reasonable cost as they pay more attention to the service quality and service perception of the medical behavior itself. Seven models including XGBoost, SVM, RF, and NNAR were proposed to predict patient flow over the next 14 days and 12 months.

The analysis and prediction results obtained from the prediction model show that the obstetric inpatient flow is not a purely random process and that the patient flow is not only accompanied by the random patient flow but also presents the trend and seasonal changes. The trend change law is increasing year by year. The seasonal variation law is that the patient flow presents the trough in the first and fourth quarters of each year, and reaches the peak in the second and third quarters. The flow of patients shows a trough from Friday to Sunday and peaks from Monday to Thursday. The distribution of random flow is approximately normal.

The HoltWinters model is used to predict the monthly flow of patients over the next year. ACF, PACF, Ljung_box and residual histogram are used to verify the accuracy of the prediction model, and the results show that the Holtwiners model has reached the optimal level. Combined with the prediction of patient flow, a more reasonable and fair horizontal allocation of medical resources is proposed.

Seven models such as XGBoost, SVM, RF, and NNAR are used to predict the daily patient flow in the next 14 days. R2, MAPE, and other indicators were used to measure the accuracy of the 14-day prediction model. The results showed that Holtwiners and STL prediction models achieved high accuracy.

This method is helpful for optimizing the allocation of medical resources, reducing medical costs, and improving service efficiency and quality.

## Code Availability

All code is open source with no restrictions and is available from https://github.com/hometownjlu/obstetric-patient-flow.

## Data Availability Statement

The datasets presented in this study can be found in online repositories. The names of the repository/repositories and accession number(s) can be found below: https://github.com/hometownjlu/obstetric-patient-flow.

## Author Contributions

HL, DM, and DW designed and planned the study. HL, PW, and YL implemented the study and drafted the article. PW and YL analyzed and validated the data under the supervision of DM and DW. All authors participated in data analysis and result interpretation, carefully revised all contents of the manuscript, and critically reviewed and approved the submitted manuscript.

## Funding

This work was supported by the National Natural Science Foundation of China (71974074), the Science and Technology Development Plan of Jilin Province (20200301004RQ), and the Education Department of Jilin Province (20211103KJ).

## Conflict of Interest

The authors declare that the research was conducted in the absence of any commercial or financial relationships that could be construed as a potential conflict of interest.

## Publisher's Note

All claims expressed in this article are solely those of the authors and do not necessarily represent those of their affiliated organizations, or those of the publisher, the editors and the reviewers. Any product that may be evaluated in this article, or claim that may be made by its manufacturer, is not guaranteed or endorsed by the publisher.
