# Supplementary material for: Prediction of Obstetric Patient Flow and Horizontal Allocation of Medical Resources Based on Time Series Analysis
Source: Front Public Health. 2021 Oct 14;9:646157. doi: 10.3389/fpubh.2021.646157 (PMC8562385; doi:10.3389/fpubh.2021.646157)
Supplement: Supplementary file 1 [file Appendix.docx]

Appendix 1. All abbreviations

| Name | Mean |
| --- | --- |
| ME | Mean Error |
| RMSE | Root Mean Squared Error |
| MAE | Mean Absolute Error |
| MPE | Mean Percentage Error |
| MAPE | Mean Absolute Percentage Error |
| ACF1 | Autocorrelation of errors at lag 1 |

Appendix 2. Hyperparameters of seven models

Nnar: Average of 20 networks, each of which is a 6-4-1 network with 33 weights options were - linear output units

HoltWinters:

Smoothing parameters: alpha: 0.03076294, beta : 0.0612446, gamma: 0.03917328

Coefficients:[,1],a 29.34389745,b 0.03170743,s1 11.01466031,s2 1.43413921

s3 1.62333880,s4 1.95730726,s5 -0.50240599,s6 -7.03734238,s7 -5.21173090

STLM：Coefficients:

1 2 3 4 5

0.1123 -0.1037 -0.0209 -0.0302 0.2117

Order selected 5 sigma^2 estimated as 45.87

ARIMA(0,0,0)(2,0,0)[7] with non-zero mean

Box Cox transformation: lambda= 0

Coefficients:

sar1 sar2 mean

0.3135 0.1380 3.2931

s.e. 0.0845 0.0867 0.0455

sigma^2 estimated as 0.09839: log likelihood=-35.45

AIC=78.9 AICc=79.2 BIC=90.67

xgb.attributes: niter.callbacks: cb.evaluation.log().# of features: 20.niter: 26.

nfeatures : 20

evaluation_log: iter train_rmse 1.21.348457,2.15.913761,--25.0.279223,26. 0.255718

RandomForest:Regressor(bootstrap=True, criterion='mse', max_depth=None, max_features='auto', max_leaf_nodes=None, min_impurity_decrease=0.0, min_impurity_split=None, min_samples_leaf=1, min_samples_split=2, min_weight_fraction_leaf=0.0, n_estimators=10, n_jobs=1, oob_score=False, random_state=None, verbose=0, warm_start=False)

SVR:(C=1.0, cache_size=200, coef0=0.0, degree=3, epsilon=0.1, gamma=0.1,

kernel='rbf', max_iter=-1, shrinking=True, tol=0.001, verbose=False)
